# Mortality Among People Experiencing Homelessness in San Francisco During the COVID-19 Pandemic

**DOI:** 10.1001/jamanetworkopen.2022.1870

**Published:** 2022-03-10

**Authors:** Caroline Cawley, Hemal K. Kanzaria, Barry Zevin, Kelly M. Doran, Margot Kushel, Maria C. Raven

**Affiliations:** 1 Department of Emergency Medicine, University of California, San Francisco; 2 Benioff Homelessness and Housing Initiative, Center for Vulnerable Populations, University of California, San Francisco; 3 Philip R. Lee Institute for Health Policy Studies, University of California, San Francisco; 4 Street Medicine and Shelter Health, San Francisco Department of Public Health, San Francisco, California; 5 Departments of Emergency Medicine and Population Health, New York University Grossman School of Medicine, New York

## Abstract

**Importance:**

There has been recent media attention on the risk of excess mortality among homeless individuals during the COVID-19 pandemic, yet data on these deaths are limited.

**Objectives:**

To quantify and describe deaths among people experiencing homelessness in San Francisco during the COVID-19 pandemic and to compare the characteristics of these deaths with those in prior years.

**Design, Setting, and Participants:**

A cross-sectional study tracking mortality among people experiencing homelessness from 2016 to 2021 in San Francisco, California. All deceased individuals who were homeless in San Francisco at the time of death and whose deaths were processed by the San Francisco Office of the Chief Medical Examiner were included. Data analysis was performed from August to October 2021.

**Exposure:**

Homelessness, based on homeless living status in an administrative database.

**Main Outcomes and Measures:**

Descriptive statistics were used to understand annual trends in demographic characteristics, cause and manner of death (based on autopsy), substances present in toxicology reports, geographic distribution of deaths, and use of health and social services prior to death. Total estimated numbers of people experiencing homelessness in San Francisco were assessed through semiannual point-in-time counts. The 2021 point-in-time count was postponed owing to the COVID-19 pandemic.

**Results:**

In San Francisco, there were 331 deaths among people experiencing homelessness in the first year of the COVID-19 pandemic (from March 17, 2020, to March 16, 2021). This number was more than double any number in previous years (eg, 128 deaths in 2016, 128 deaths in 2017, 135 deaths in 2018, and 147 deaths in 2019). Most individuals who died were male (268 of 331 [81%]). Acute drug toxicity was the most common cause of death in each year, followed by traumatic injury. COVID-19 was not listed as the primary cause of any deaths. The proportion of deaths involving fentanyl increased each year (present in 52% of toxicology reports in 2019 and 68% during the pandemic). Fewer decedents had contacts with health services in the year prior to their death during the pandemic than in prior years (13% used substance use disorder services compared with 20% in 2019).

**Conclusions and Relevance:**

In this cross-sectional study, the number of deaths among people experiencing homelessness in San Francisco increased markedly during the first year of the COVID-19 pandemic. These findings may guide future interventions to reduce mortality among individuals experiencing homelessness.

## Key Points

**Question:**

How did the number and characteristics of deaths among people experiencing homelessness in San Francisco during the COVID-19 pandemic compare with those in prior years?

**Findings:**

In this cross-sectional study, more than twice as many people died while homeless in the year starting March 17, 2020, compared with any prior year.

**Meaning:**

The number of deaths in San Francisco among people experiencing homelessness increased markedly during the COVID-19 pandemic, with most of the increase associated with overdose deaths rather than COVID-19 itself.

## Teaser

This cross-sectional study quantifies and describes deaths among people experiencing homelessness in San Francisco during the COVID-19 pandemic and compares the characteristics of these deaths with those in prior years.

## Introduction

San Francisco has been tracking deaths among people experiencing homelessness using standardized methods since 2016.^1^ Early in the COVID-19 pandemic, there was concern about the potential for high rates of COVID-19 transmission and mortality among people experiencing homelessness.^2^ To mitigate this risk, San Francisco placed several thousand people experiencing homelessness at high risk of severe complications from COVID-19 in shelter-in-place hotel rooms, reduced the number of people in shelters, and offered mitigation strategies in sheltered and unsheltered settings.^3^ In this study, we describe deaths among people experiencing homelessness in the first year of the pandemic (from March 17, 2020, to March 16, 2021) and compare the characteristics of these deaths with those of the deaths in 4 preceding calendar years. We sought to understand the factors associated with mortality among people experiencing homelessness and to inform mortality prevention strategies.

## Methods

In this cross-sectional study, to assess the number of deaths among people experiencing homelessness in San Francisco, California, we reviewed records from the San Francisco Office of the Chief Medical Examiner (OCME).^4^ The OCME provides forensic death investigation services for the City and County of San Francisco and generates reports providing manner, cause, location, and date of death. Cases also include autopsy and toxicology reports, except in situations involving a lengthy hospital stay prior to death because, when measured weeks into a hospitalization, postmortem toxicology would not accurately reflect substances that may have been present at the time of admission. We linked these data to the San Francisco Department of Public Health (SFDPH) Coordinated Care Management System (CCMS) to obtain data on demographic characteristics, homelessness history, and the use of medical services, behavioral health services, county jail health services, shelter-in-place hotels, and shelters prior to death for each decedent. The CCMS defines homelessness based on either data on homeless service use or data on self-reported homeless status collected during health or social service encounters. The SFDPH creates a CCMS record for any individual noted as homeless in any San Francisco County health or housing data system, any individual using county behavioral health, homelessness, or jail health services, or any individual using county urgent or emergent medical, mental health, or substance use services. Descriptions of these data sources can be found elsewhere.^1,5^ We categorized decedents as homeless if they met at least 1 criterion for the US Department of Housing and Urban Development definition of homelessness.^6^ We obtained institutional review board approval from the University of California, San Francisco and adhered to the Protected Health Information and Code of Federal Regulations 42 part 2 protocols governing the use of substance use disorder treatment data. Informed consent was not provided because the study did not involve contact with individuals (all individuals were deceased). Our report follows the Strengthening the Reporting of Observational Studies in Epidemiology (STROBE) reporting guideline for observational cross-sectional studies.

### Statistical Analysis

We calculated homeless death estimates for a 12-month period starting March 17, 2020 (the date of San Francisco’s shelter-in-place order), and compared these estimates with the annual estimates for the calendar years 2016 to 2019. We expressed estimates as rates per 100 000 based on semiannual point-in-time counts of homelessness^7^ to account for relative changes in the homeless population over time. For example, there were 6858 individuals in 2017 and 8035 individuals in 2019. The 2021 point-in-time count was postponed owing to the COVID-19 pandemic. We used descriptive statistics (SAS, version 9.4 [SAS Institute]) to understand trends in demographic characteristics, living situations, causes and locations of death, toxicology findings, and use of health and social services in the year prior to death.

## Results

**Figure 1.**
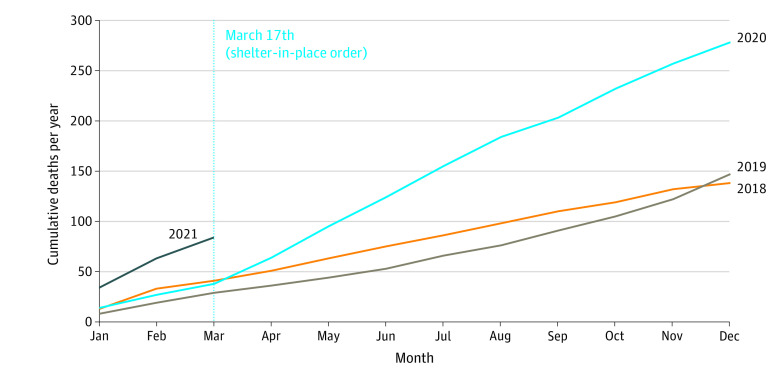
Cumulative Annual Deaths Among People Experiencing Homelessness in San Francisco From January 1, 2018, to March 16, 2021

**Figure 2.**
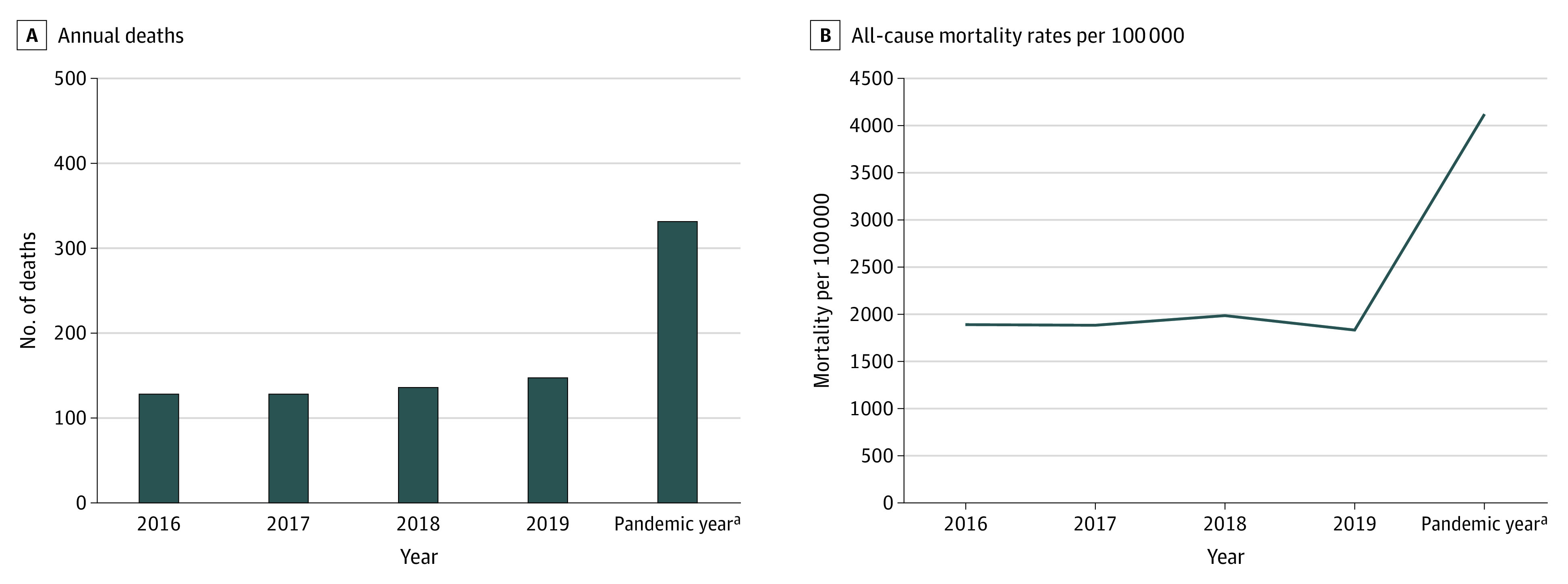
Annual Deaths and All-Cause Mortality Rates per 100 000 Among People Experiencing Homelessness in San Francisco From January 1, 2016, to March 16, 2021 ^a^From March 17, 2020, to March 16, 2021.

In the first year of the pandemic (from March 17, 2020, to March 16, 2021), there were 331 deaths among people experiencing homelessness, more than double the annual total of any previous year (128 in 2016, 128 in 2017, 135 in 2018, and 147 in 2019). There was an inflection point of increased mortality shortly after shelter-in-place began (Figure 1). The all-cause mortality rate also doubled, from 1829 per 100 000 in 2019 to 4119 per 100 000 in the first year of the pandemic (Figure 2). The demographic characteristics of decedents remained similar across study years (Table 1) and were similar to the general homeless population in San Francisco; most were men aged 40 to 60 years, had been experiencing homelessness for at least 5 years, and disproportionately identified as African American or Black (22%-31%) relative to the general population of San Francisco (5.6%).^8^

**Table 1.** Characteristics of Deaths Among People Experiencing Homelessness in San Francisco, by Year

Characteristic	Decedents, No. (%)				
	2016 (n = 128)	2017 (n = 128)	2018 (n = 135)	2019 (n = 147)	Pandemic year (n = 331)^a^
Age at death, median (range), y	49 (21-86)	54 (24-80)	52 (22-80)	46 (18-86)	47 (0-77)
Gender					
Female	24 (19)	16 (13)	18 (13)	24 (16)	62 (19)
Male	104 (81)	111 (87)	117 (87)	123 (84)	268 (81)
Transgender	0	1 (1)	0	0	1 (<1)
Race and ethnicity					
African American or Black	33 (26)	39 (30)	30 (22)	46 (31)	90 (27)
Asian	2 (2)	3 (2)	6 (4)	7 (5)	20 (7)
Latino or Latina	15 (12)	16 (13)	16 (12)	17 (12)	51 (15)
Mixed or other^b^	4 (3)	3 (2)	4 (3)	2 (2)	10 (3)
White	70 (55)	66 (52)	74 (55)	72 (49)	159 (48)
Unknown	4 (3)	1 (<1)	5 (4)	3 (2)	1 (<1)
Length of time homeless in San Francisco, y					
<1	13 (14)	15 (15)	15 (13)	26 (20)	63 (22)
1 to <5	20 (22)	25 (26)	28 (25)	32 (25)	55 (19)
5 to <10	25 (27)	12 (12)	16 (14)	14 (11)	52 (18)
≥10	35 (38)	45 (46)	53 (47)	56 (44)	113 (40)

^a^ From March 17, 2020, to March 16, 2021.

^b^ Includes the response options mixed, multiethnic, other, and Native American.

The proportion of deaths due to overdose increased since we began gathering data (34% [39 of 116] in 2016, 34% [36 of 107] in 2017, 56% [66 of 118] in 2018, and 72% [98 of 137] in 2019); that trend continued during the COVID-19 pandemic, reaching 82% (178 of 216). Traumatic injuries (including homicides and suicides) were the second leading cause of death each year (Table 2), followed by chronic medical conditions (eg, cardiovascular disease and cancer). All decedents were tested for COVID-19. Four individuals tested positive either post mortem or during the hospital admission preceding their death, but COVID-19 was not listed as the primary cause of death in any of these cases. Deaths involving fentanyl increased throughout the study period. In 2016 and 2017, there were few fentanyl-involved cases (6% [6 of 97] and 7% [7 of 96] of toxicology reports, respectively), increasing to 30% (32 of 106) of toxicology reports in 2018, 52% (67 of 128) in 2019, and 68% (144 of 213) during the pandemic year. In every year, methamphetamines were the most common substances present in toxicology reports (48% [47 of 97] in 2016, 46% [44 of 96] in 2017, 66% [70 of 106] in 2018, 79% [101 of 128] in 2019, and 77% [164 of 213] during the pandemic).

**Table 2.** Causes of Death Among People Experiencing Homelessness in San Francisco, by Year

Cause of death	Decedents, No. (%)				
	2016 (n = 116)	2017 (n = 107)	2018 (n = 118)	2019 (n = 137)	Pandemic year (n = 216)^a^
Acute drug toxicity	39 (34)	36 (34)	66 (56)	98 (72)	178 (82)
Traumatic injury	31 (27)	32 (30)	24 (20)	14 (10)	14 (6)
Chronic medical conditions	28 (25)	27 (26)	18 (15)	12 (9)	12 (5)
Complications of chronic alcohol use	12 (10)	5 (5)	6 (5)	7 (5)	7 (3)
Other	6 (5)	7 (7)	4 (3)	6 (4)	5 (2)

^a^ From March 17, 2020, to March 16, 2021.

Of the 869 deaths, 442 (51%) occurred outdoors (eg, sidewalk and park) in all study years. There were 54 deaths in which the death or precipitating incident occurred in shelter-in-place hotel rooms (eg, overdose in shelter-in-place room followed by death while hospitalized), plus an additional 16 shelter-in-place guests who died elsewhere (eg, friend’s residence). The all-cause mortality rate for shelter-in-place guests was 1811 per 100 000 (based on the alternate shelter program having served 3864 guests in shelter-in-place hotels or trailers^3^) compared with an estimated 5038 per 100 000 for non–shelter-in-place guests (based on the most recent point-in-time count of unsheltered individuals in San Francisco).

More than 90% of decedents in each year were successfully matched to CCMS, indicating that they were previously known to San Francisco public health and social services. Among those who died in the pandemic year, two-thirds (64% [212 of 331]) used medical services (acute care and/or outpatient [eg, emergency departments and SFDPH primary care clinics]) in the 12 months prior to death compared with 76% (112 of 147) in 2019. Of 331 decedents, 79 (24%) had contact with mental health services (eg, psychiatric emergency services and behavioral health case management), and 43 (13%) had contact with substance use disorder services (eg, sobering center and residential detoxification), compared with 30% (44 of 147) and 20% (30 of 147), respectively, in 2019 in the 12 months prior to death. Of 331 decedents, 86 (26%) had been in county jail custody in the year prior to death (compared with 35% [52 of 147] in 2019).

## Discussion

Deaths among people experiencing homelessness in San Francisco more than doubled to 331 deaths during the first year of the COVID-19 pandemic, driven by a large increase in overdose deaths. No deaths in our data set were due to COVID-19 itself, which may speak to the success of San Francisco’s efforts to mitigate the spread of the virus in vulnerable populations, including by moving individuals at high risk into noncongregate settings. However, the pandemic had far-reaching effects on outreach, health, and social services (eg, temporary closures or reduced hours for drop-in behavioral health facilities; decreased capacity in residential facilities to allow social distancing; and staff redeployments to COVID-19–related roles) that may have contributed to increased mortality from non–COVID-19 causes. In a study of women who were homeless or unstably housed in San Francisco, some study participants reported increased difficulties getting care for chronic medical conditions, mental health, and substance use during the pandemic.^9^ The general population of the US has also experienced an increase in excess deaths not attributed to COVID-19 during the pandemic.^10^

This disruption to services coincided with increasing unintended overdose deaths in San Francisco, driven by the growing presence of fentanyl. There was a continued increase in the proportion of deaths among people experiencing homelessness associated with overdoses compared with 2019 (72% in 2019 and 82% in the study year), which followed a years-long upward trend, and the absolute number of overdose deaths increased markedly. From 2018 to 2019, the Western US experienced a large increase in death rates involving synthetic opioids, such as fentanyl.^11^ In Boston, Massachusetts, the synthetic opioid mortality rate among people experiencing homelessness increased by more than 1400% between 2013 and 2018. This homeless cohort’s standardized mortality rate from overdose for the years 2003 to 2018 was also 12 times higher than that of the adult population of Massachusetts.^12,13^ A study of total overdose deaths in San Francisco found a 50% increase in weekly median overdose deaths since the onset of the pandemic.^14^

COVID-19 mitigation policies may have affected how, when, and where people used drugs and the chances a passerby could intervene in case of an overdose. COVID-19–related effects on the health system may have led to decreased access to treatment. We found that a low proportion of decedents had recent contact with behavioral health services, including office-based substance use disorder treatment programs. Our data on substance use services do not capture information on all of San Francisco’s overdose prevention programs (eg, naloxone distribution), but our findings point to a need for more programs to address substance use disorder and overdose risk among people experiencing homelessness. San Francisco’s alternative shelter program (shelter-in-place hotels) did not appear to be associated with increased overdose deaths among people experiencing homelessness, as some had feared at the start of the program.^15^ The all-cause and overdose-specific mortality rates were lower for shelter-in-place guests than for other people experiencing homelessness, despite their targeting individuals at higher risk of mortality.

Of note, our findings stand in contrast to those elsewhere in the country. For example, in New York City in fiscal year 2020, COVID-19 was the second leading cause of death (after overdose) among people experiencing homelessness (121 deaths through June 30, 2020).^16^ While there is no standard tracking of deaths among people experiencing homelessness across the US, 1 grassroots effort has compiled publicly available documentation to discover that there have been at least 553 deaths due to COVID-19 among people experiencing homelessness across 23 US cities and counties.^17^

### Limitations

The limitations of this study include our lack of data on non-OCME cases and reliance on point-in-time data to estimate people experiencing homelessness.^18^ Through their mandate to process all sudden, unexpected, violent, unobserved, overdose, and indigent deaths in San Francisco, the OCME handles most, but not all, deaths among people experiencing homelessness.

There are some system touchpoints not captured by the CCMS, including substance use harm reduction services. Considering the very high prevalence of substance-involved deaths in our study population, having these data would provide important details on additional service encounters prior to death. The CCMS also does not include medical records from certain non-SFDPH health care facilities (eg, Veterans Health Administration records) and does not capture homelessness history from other regions.

## Conclusions

Our findings highlight the importance of tracking deaths among people experiencing homelessness. Examining the cause of death and the location of decedents is critical; without these data, one might have assumed that the increased deaths in the pandemic year were due to COVID-19 infection. Finally, having mortality data linked with other service use data (eg, health care services) allows for the identification of potential gaps and points of intervention to prevent deaths.
